# A versatile approach to high-density microcrystals in lipidic cubic phase for room-temperature serial crystallography

**DOI:** 10.1107/S1600576723006428

**Published:** 2023-08-18

**Authors:** James Birch, Tristan O. C. Kwan, Peter J. Judge, Danny Axford, Pierre Aller, Agata Butryn, Rosana I. Reis, Juan F. Bada Juarez, Javier Vinals, Robin L. Owen, Eriko Nango, Rie Tanaka, Kensuke Tono, Yasumasa Joti, Tomoyuki Tanaka, Shigeki Owada, Michihiro Sugahara, So Iwata, Allen M. Orville, Anthony Watts, Isabel Moraes

**Affiliations:** a Membrane Protein Laboratory, Diamond Light Source, Harwell Science and Innovation Campus, Didcot, Oxfordshire OX11 0DE, United Kingdom; b Research Complex at Harwell, Rutherford Appleton Laboratory, Harwell Science and Innovation Campus, Didcot, Oxfordshire OX11 0FA, United Kingdom; c ChemBio, National Physical Laboratory, Hampton Road, Teddington, Middlesex TW11 0LW, United Kingdom; d Biochemistry Department, Oxford University, South Parks Road, Oxford OX1 3QU, United Kingdom; e Diamond Light Source, Harwell Science and Innovation Campus, Didcot, Oxfordshire OX11 0DE, United Kingdom; f Ecole Polytechnique Fédérale de Lausanne (EPFL), Station 19, Lausanne, CH-1015, Switzerland; g Department of Biological Chemistry and Molecular Pharmacology, Harvard Medical School, Boston, Massachusetts 02115, USA; h RIKEN SPring-8 Center, 1-1-1 Kouto, Sayo-cho, Sayo-gun, Hyogo, 679-5148, Japan; i Institute of Multidisciplinary Research for Advanced Materials, Tohoku University, 2-1-1 Katahira, Aoba-ku, Sendai, 980-8577, Japan; j Department of Cell Biology, Graduate School of Medicine, Kyoto University, Yoshidakonoe-cho, Sakyo-ku, Kyoto, 606-8501, Japan; k Japan Synchrotron Radiation Research Institute, 1-1-1 Kouto, Sayo-cho, Sayo-gun, Hyogo, 679-5148, Japan; University of Michigan, USA

**Keywords:** serial crystallography, membrane proteins, lipidic cubic phase, archaerhodopsin-3, A_2A_ adenosine receptor, structure-based drug design

## Abstract

Serial crystallography has emerged as an important tool for structural studies of integral membrane proteins. The ability to collect data from micrometre-sized weakly diffracting crystals at room temperature with minimal radiation damage has opened many new opportunities in time-resolved studies and drug discovery. However, the production of integral membrane protein microcrystals in lipidic cubic phase at the desired crystal density and quantity is challenging. This paper introduces VIALS (versatile approach to high-density microcrystals in lipidic cubic phase for serial crystallography), a simple, fast and efficient method for preparing hundreds of microlitres of high-density microcrystals suitable for serial X-ray diffraction experiments at both synchrotron and free-electron laser sources. The method is also of great benefit for rational structure-based drug design as it facilitates *in situ* crystal soaking and rapid determination of many co-crystal structures. Using the VIALS approach, room-temperature structures are reported of (i) the archaerhodopsin-3 protein in its dark-adapted state and 110 ns photocycle intermediate, determined to 2.2 and 1.7 Å, respectively, and (ii) the human A_2A_ adenosine receptor in complex with two different ligands determined to a resolution of 3.5 Å.

## Abstract 2

A versatile approach for the preparation of high-density membrane protein microcrystals in lipidic cubic phase for serial crystallography is described.

## 1. Introduction

Like any other experimental technique, X-ray crystallography has evolved and matured, and has re-invented itself over the years. The advent of ultrafast high-brilliance X-ray sources such as free-electron lasers (XFELs) and the latest-generation synchrotrons, in addition to technical developments in continuous sample delivery (Botha *et al.*, 2015 ▸; Grünbein & Kovacs, 2019 ▸; Kubo *et al.*, 2017 ▸; Lomb *et al.*, 2012 ▸; Martin-Garcia *et al.*, 2019 ▸; Shimazu *et al.*, 2019 ▸; Tono *et al.*, 2015 ▸; Weierstall *et al.*, 2012 ▸, 2014 ▸; Zielinski *et al.*, 2022 ▸), new fast-readout and low-noise detectors (Henrich *et al.*, 2011 ▸; Kameshima *et al.*, 2014 ▸; Kubo *et al.*, 2017 ▸; Leonarski *et al.*, 2018 ▸), and new software capable of processing large amounts of data (Grosse-Kunstleve *et al.*, 2002 ▸; Kabsch, 2014 ▸; Kirian *et al.*, 2011 ▸; Nakane *et al.*, 2016 ▸; White *et al.*, 2013 ▸, 2016 ▸; Winter *et al.*, 2018 ▸), have enabled the advancement of room-temperature serial crystallography (SX) (Barends *et al.*, 2022 ▸; Boutet *et al.*, 2012 ▸; Chapman *et al.*, 2011 ▸; Dods *et al.*, 2021 ▸; Moreno-Chicano *et al.*, 2019 ▸; Neutze *et al.*, 2000 ▸; Orville, 2020 ▸).

Room-temperature SX at XFELs (SFX) and synchrotron sources (SSX) is applicable to membrane protein samples crystallized in lipidic cubic phase (LCP) (Caffrey & Cherezov, 2009 ▸; Liu *et al.*, 2014 ▸). High-resolution structural data generated for integral membrane proteins (IMPs) (Axford *et al.*, 2022 ▸; Johansson *et al.*, 2019 ▸; Hosaka *et al.*, 2022 ▸; Liu *et al.*, 2013 ▸; Nango & Iwata, 2023 ▸; Nogly *et al.*, 2015 ▸; Stauch *et al.*, 2019 ▸; Zhang *et al.*, 2017 ▸, to mention a few) are critical for understanding structure–function relationships, intermolecular interactions and protein dynamics, and for rational drug design (Hauser *et al.*, 2018 ▸; Reis & Moraes, 2019 ▸). Structure determination of IMPs by traditional X-ray diffraction methods is challenging, since the crystals are usually extremely fragile and exceptionally sensitive to radiation damage. Additionally, the cryogenic temperatures typically required restrict the conformational flexibility and dynamics of biological macromolecules. SX of IMPs crystallized in LCP allows diffraction data to be acquired under near-physiological conditions, where the mesophase mimics the biological membrane. Furthermore, SX in LCP facilitates time-resolved studies, critical for generating mechanistic insights into the dynamic behaviour of IMPs (Hosaka *et al.*, 2022 ▸; Mous *et al.*, 2022 ▸ *;* Nango *et al.*, 2016 ▸; Nass Kovacs *et al.*, 2019 ▸; Nogly *et al.*, 2016 ▸, 2018 ▸; Oda *et al.*, 2021 ▸; Skopintsev *et al.*, 2020 ▸; Weinert *et al.*, 2019 ▸; Yun *et al.*, 2021 ▸).

Although SX of IMPs crystallized in LCP has been successful in overcoming many of the challenges associated with classical diffraction methods, technical and practical difficulties remain. SX methods typically consume large amounts of protein (up to several milligrams) and the crystals must be in a narrow size range (1–5 µm) and grow at sufficiently high density to maximize the efficiency of data collection. This is particularly important during time-resolved experiments, which require the collection of complete data sets for multiple time points (Brändén & Neutze, 2021 ▸; Grünbein *et al.*, 2020 ▸). In addition, crystallization of IMPs in LCP for SX requires large numbers of gas-tight glass syringes, which are also needed during the screening process for optimizing crystallization conditions (Liu *et al.*, 2014 ▸). Failure to seal or store these syringes correctly results in sample leakage or dehydration. Furthermore, transport of samples to X-ray facilities can be associated with glass breakage and sample loss.

**Figure 1:**
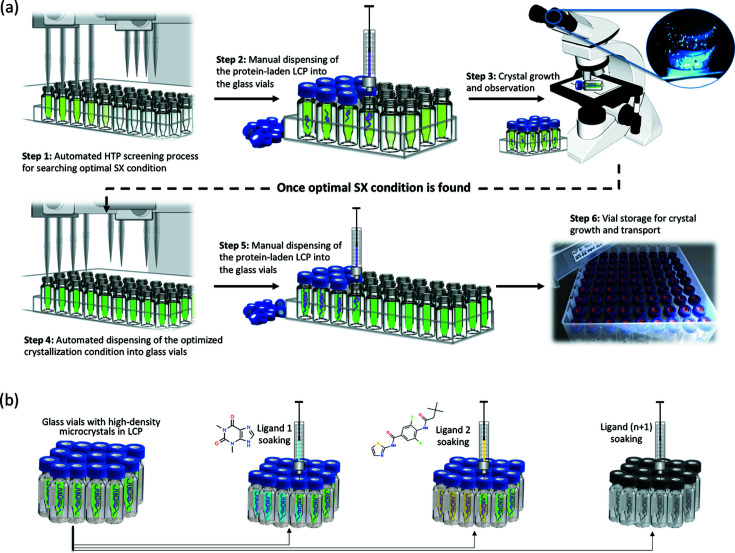
Schematic representation of the VIALS workflow. (*a*) Flowchart depicting the semi-automated high-throughput procedure of VIALS. Step 1: design and automated preparation of a rational grid screen based on a previously known crystallization condition (*e.g.* for the growth of large crystals). Step 2: tiny amounts of protein-laden LCP string are manually injected into each previously prepared small glass vial. Step 3: periodic direct inspection of crystal growth using a stereo high-magnification microscope. Steps 4–5: scale-up process once the optimal microcrystal size/density condition is found. Step 6: microcrystal growth and storage for SX measurements. (*b*) VIALS experimental setup for ligand soaking *in meso* in parallel ahead of room-temperature SX data collection.

To address the above issues inherent in current methods, we have developed an alternative approach that does not require the use of large quantities of gas-tight syringes for the production of a sufficient amount of homogeneous crystals in LCP for SX studies. The procedure, termed VIALS (versatile approach to high-density microcrystals in lipidic cubic phase for serial crystallography), makes use of small borosilicate glass vials (sample volume of 300 µl) and an automated liquid-handler system to produce hundreds of microlitres of high-density micrometre-sized crystals grown in LCP [Fig. 1 ▸(*a*)]. The approach also facilitates ligand soaking without mechanically disturbing the crystals. Ligand soaking, vital to rational structure-based drug design, has been particularly challenging when growing IMP crystals in LCP within gas-tight glass syringes (Ishchenko *et al.*, 2019 ▸; Weinert *et al.*, 2017 ▸). The method introduced here enables soaking of many ligands in parallel, facilitating structure determination of ligand–receptor complexes in a single SX experimental session [Fig. 1 ▸(*b*)]. The VIALS workflow is simple, fast and economical, and is easily implemented in any standard crystallization laboratory. Finally, storage and transport of crystal samples in LCP are also facilitated by our approach [Fig. 1 ▸(*a*), step 6].

To validate the versatility of the VIALS workflow, we used two different IMPs, archaerhodopsin-3 (AR3) and the human adenosine A_2A_ receptor (hA_2A_R), as proof of concept. AR3, from the archaebacterium *Halorubrum sodomense*, is a light-driven proton pump that undergoes a sequence of conformational changes induced by the absorption of a photon and is commonly used in optogenetic experiments (Bada Juarez *et al.*, 2021 ▸). hA_2A_R is a class A G-protein-coupled receptor that is expressed in the cardiovascular, respiratory, immune and central nervous systems, and is implicated in immunosuppression and the regulation of sleep (Fredholm *et al.*, 2001 ▸).

Here we report two room-temperature AR3 structures: the first, by SSX, is the dark-adapted state of the protein; the second, by time-resolved SFX, is the 110 ns photocycle intermediate. We also report crystal structures of human hA_2A_R in complex with two different ligands determined by SSX.

## 2. Materials and methods

### 2.1. Protein production and reconstitution in LCP

The AR3 protein was produced as previously described (Bada Juarez *et al.*, 2021 ▸). The purified protein sample was concentrated in distilled water to 20 mg ml^−1^ and stored in the dark at 4°C. AR3 was reconstituted in LCP at 20°C, by mixing the protein with monoolein lipid [9.9 mono­acyl­glycerol (MAG), Nu-Chek Prep Inc.] in a 40:60 volume ratio using two 100 µl gas-tight Hamilton syringes (No. 81065, Hamilton) connected by a syringe coupler (SPTLabtech), as previously described (Caffrey & Cherezov, 2009 ▸). The reconstitution procedure was performed under dim, red light.

Thermostabilized hA_2A_R was produced as previously described (Rucktooa *et al.*, 2018 ▸). The purified receptor in 40 m*M* Tris pH 7.5, 200 m*M* NaCl, 0.15%(*w*/*v*) *n*-do­decyl-β-maltoside, 70 m*M* imidazole and 1 m*M* theophylline (a low-affinity antagonist) was concentrated to approximately 20 mg ml^−1^ and stored at −80°C. The purified hA_2A_R was reconstituted in LCP at 20°C by mixing the protein with monoolein lipid, supplemented with 10%(*w*/*w*) cholesterol (Sigma Aldrich) in a 40:60 volume ratio, using the twin-syringe method (Caffrey & Cherezov, 2009 ▸).

### 2.2. Production of high-density micrometre-sized crystals using the VIALS approach

#### 2.2.1. Archaerhodopsin-3

Starting with a previously known crystallization condition for the growth of large crystals (>40 µm), a grid screen of solutions composed of 28–36%(*v*/*v*) polyethyl­ene glycol 600 (PEG 600) (Fluka Analytical), 100 m*M* MES buffer pH 5.0–6.5, 150 m*M* NaCl and 50–200 m*M* CaCl_2_ was prepared and dispensed by an automated liquid-handler system (Hamilton StarLet) into glass vials purchased from Thermo Scientific (catalogue No. 17324073) [Fig. 1 ▸(*a*), step 1]. Approximately 5–10 µl of protein-laden LCP was injected (using a gas-tight Hamilton syringe of 100 µl connected to a SPTLabtech mosquito LCP narrow-bore short needle of 0.15 mm internal diameter) into each small glass vial filled with 300 µl of precipitant screen solution, and each vial was sealed with a screw cap also purchased from Thermo Scientific (catalogue No. 17334043) [Fig. 1 ▸(*a*), step 2]. The crystallization procedure was performed under dim, red light and the glass vials were stored in storage boxes at 20°C in the dark. Crystal growth was periodically monitored using a stereo high-magnification microscope equipped with cross-polarizers [Fig. 1 ▸(*a*), step 3]. Occasionally, a small amount of crystal-laden LCP sample was retrieved, sandwiched between two glass slides, and observed under the microscope with the help of the dark-field and cross-polarizer [Fig. S1(*a*) in the supporting information]. Large quantities of AR3 micrometre-sized crystals in LCP appeared after 2 to 3 days, with their density and size varying as a function of PEG 600 and CaCl_2_ concentration. Ahead of the SSX and SFX measurements, many glass vials containing the same optimized crystallization solution [33%(*v*/*v*) PEG 600, 100 m*M* MES buffer pH 5.5, 150 m*M* NaCl and 150 m*M* CaCl_2_] were prepared, each containing ∼30–50 µl of protein-laden LCP thread [Fig. 1 ▸(*a*), steps 4–6, and Fig. S1].

#### 2.2.2. Human A_2A_ adenosine receptor

Following the VIALS protocol as described above (see Section 2.2.1), around 5–10 µl of hA_2A_R reconstituted in LCP was dispensed into each glass vial previously filled with 300 µl of precipitant solution composed of 29–32% polyethyl­ene glycol 400 (PEG 400) (Fluka Analytical), 0.1 *M* tri-sodium citrate pH 4.0–5.5, 0.05 *M* sodium thio­cyanate and 2%(*v*/*v*) 2,5-hexane­diol to identify the best crystallization condition associated with high microcrystal density [Fig. 1 ▸(*a*), steps 1–2]. The glass vials were stored at 20°C and crystal growth periodically inspected as described above (Section 2.2.1). High-density micrometre-sized crystals of hA_2A_R appeared within a few days, and the crystal density and size varied as a function of PEG 400 concentration and tri-sodium citrate pH (Fig. S1). Prior to SSX beam time, many glass vials containing the same optimized crystallization solution, 0.1 *M* tri-sodium citrate pH 4.5, 0.05 *M* sodium thio­cyanate, 29%(*v*/*v*) PEG 400 and 2%(*v*/*v*) 2,5-hexane­diol [Fig. 1 ▸(*a*), steps 4–6], were prepared and stored at 20°C.

### 2.3. Room-temperature SSX data collection

SSX diffraction data were collected at 20°C on the I24 beamline at Diamond Light Source (DLS) using a high-viscosity LCP extruder installed vertically at 90° to the synchrotron X-ray beam [Fig. S2(*a*)]. The LCP extruder was operated using a high-performance liquid chromatography water pump and compressed helium gas as described by Weierstall *et al.* (2014 ▸). High-density AR3 and hA_2A_R microcrystals of size 5–10 µm in LCP threads were retrieved from the glass vials using a clean, gas-tight Hamilton syringe plunger and transferred to a 250 µl gas-tight Hamilton syringe (No. 81120 Hamilton). This was to remove the excess mother liquor (Fig. S3) and facilitate sample loading into the LCP extruder sample reservoir of 20 µl (Weierstall *et al.*, 2014 ▸). For the AR3 crystals, the procedure above was performed under dim, red light. The AR3 and hA_2A_R crystal samples were extruded using 100 µm and 50 µm inner diameter (ID) silica capillaries, respectively, at an average pump flow rate of ∼50–150 nl min^−1^. The extruded sample was caught in a sample catcher, connected to a pump that was regularly cleaned. Data were continuously recorded (shutterless mode) by a PILATUS3 6M detector running at 10 Hz, using an X-ray beam focused at 9 × 6 µm (FWHM) with an energy of ∼12.8 keV (3.0 × 10^12^ photons s^−1^).

### 2.4. AR3 time-resolved SFX data collection

Time-resolved (TR) SFX diffraction data were collected at 20°C on the BL2 EH3 station at SPring-8 Angstrom Compact Free Electron Laser (SACLA) in Japan. AR3 crystals of around 5–10 µm in LCP threads were retrieved from the glass vials, using the same method described above (see Section 2.3 and Fig. S3), and loaded into a sample reservoir of 60 µl of a high-viscosity cartridge-type (HVC) extruder (Shimazu *et al.*, 2019 ▸; Tono *et al.*, 2015 ▸) under dim, red light. A stream of AR3 microcrystals, delivered from the HVC extruder at an average flow rate of ∼15–20 µl min^−1^ from a nozzle of 75 µm ID, was perpendicularly aligned with an XFEL beam and a nano­second synchronized optical parametric oscillator (OPO) laser in a two-way excitation open setup (Kubo *et al.*, 2017 ▸; Nango *et al.*, 2016 ▸) [Fig. S2(*b*)]. While XFEL pulses of <10 fs (440 µJ per pulse) operated at a repetition rate of 30 Hz focused into an approximately 1.5 µm (FWHM) diameter spot size, the OPO laser of 571 nm wavelength ran at 10 ns of pulse length at a frequency of 15 Hz with a focal spot of 40 µm (FWHM). The time delay between the pump laser and the X-ray pulse was 110 ns. The diffraction patterns were recorded on a 4 mega-pixel multiport charge-coupled device (4MPCCD) detector (Kameshima *et al.*, 2014 ▸) at 50 mm distance from the sample. The hit rate was continuously monitored using the SACLA real-time data-processing pipeline (Nakane *et al.*, 2016 ▸).

### 2.5. Data processing and structure determination

Diffraction patterns were integrated using *DIALS* (Winter *et al.*, 2018 ▸). Protein structures were determined by molecular replacement using *PHASER* (McCoy *et al.*, 2007 ▸) using as the search models Protein Data Bank (PDB) entries 1uaz (Enami *et al.*, 2003 ▸) for the AR3 structures and 5mzj (Cheng *et al.*, 2017 ▸) and 5olv (Rucktooa *et al.*, 2018 ▸) for the hA_2A_R structures in complex with theophylline and LUAA47070 ligands, respectively. Heteroatoms were removed from all search models. All structures were initially optimized through iterative cycles of manual rebuilding using *Coot* (Emsley *et al.*, 2010 ▸) and refinement using *PHENIX* (Adams *et al.*, 2010 ▸; Liebschner *et al.*, 2019 ▸). Ligands, lipids, water molecules and other solvent ions were later modelled on the basis of the 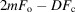 difference electron-density maps and *B* factors. For the AR3 structures, retinal occupancy ratios for both SSX and time-resolved SFX structures were determined using several tools in parallel such as occupancy refinement in *PHENIX*, observation of the retinal *B* factors before and after refinement (using different occupancy values), observation of the calculated 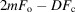 and omit maps corresponding to each occupancy value, and ligand validation tools integrated in *PHENIX* and *Coot*. For the hA_2A_R complex structures, TLS groups were initially identified using the *TLSMD* server (Painter & Merritt, 2006 ▸), with subsequent iterative cycles of restrained maximum-likelihood and TLS refinement performed using *phenix.refine*. All final structure models were validated with *MolProbity* (Chen *et al.*, 2010 ▸) implemented in *PHENIX*. Data collection and refinement statistics for the AR3 and hA_2A_R structures solved in this study are summarized in Table 1 ▸. Omit maps were generated in *PHENIX* and all the figures were prepared with *PyMOL* (Schrödinger). Structure alignments and root-mean-square deviation (RMSD) calculations were performed using *PyMOL*. The atomic coordinates and structure factors have been deposited in the PDB under the following accession codes (see Table 1 ▸ for data set names): 6guy (SSX structure of AR3_dark-adapted_); 7zy3 (time-resolved SFX structure of AR3_110 ns_); 8a2o (SSX structure of hA_2A_R–theophylline) and 8a2p (SSX structure of hA_2A_R–LUAA47070).

## 3. Results and discussion

### 3.1. Room-temperature structure of AR3 by SSX

**Figure 2:**
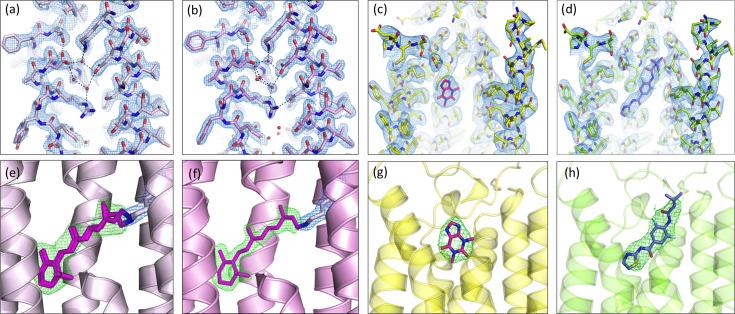
Quality of the electron-density maps. (*a*)–(*d*) 2*mF*_o_ − *DF*_c_ electron-density maps (blue mesh) contoured at the 1.5σ level showing close-up views of AR3_dark-adapted_ (PDB entry 6guy, light pink), AR3_110 ns_ (PDB entry 7zy3, light magenta), and the hA_2A_R binding site in complex with theophylline (PDB entry 8a2o, yellow) and LUAA47070 (PDB entry 8a2p, green), respectively. (*e*)–(*h*) *mF*_o_ − *DF*_c_ omit electron-density maps contoured at the ±3.0σ level showing strong positive density (green mesh) when ligands are omitted during refinement. Protein residues and ligands are shown as sticks and water molecules as red spheres. Dashed lines in panels (*a*) and (*b*) represent the hydrogen-bond network.

**Figure 3:**
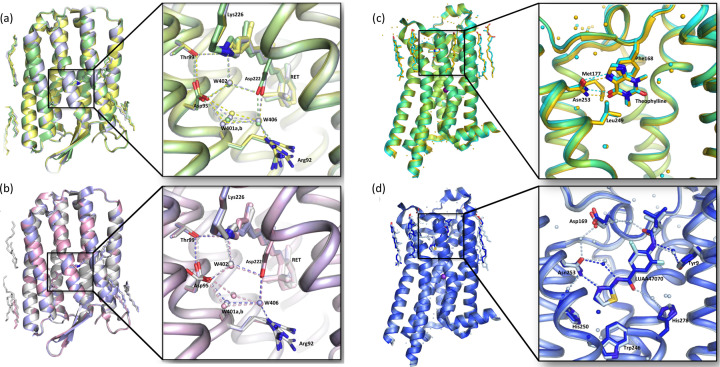
Superposition of the room-temperature SX structures with their cryogenic counterparts. (*a*) Superposition of the SSX extruder dark-adapted AR3 (PDB entry 6guy, light green), SSX dark-adapted AR3 (PDB entry 6s63, grey) and cryo dark-adapted AR3 (PDB entry 6gux, yellow) structures. The inset right panel shows details of the retinal and the pentagonal hydrogen-bond network in AR3. (*b*) Superposition of the SFX 110 ns photocycle intermediate AR3 (PDB entry 7zy3, pink), SSX light-adapted AR3 (PDB entry 6guz, purple) and cryo light-adapted AR3 (PDB entry 6s6c, silver) structures. The inset right panel shows details of the retinal and the pentagonal hydrogen-bond network in AR3. (*c*) Superposition of the SSX hA_2A_R–theophylline complex structure (PDB entry 8a2o, turquoise) and its synchrotron cryo counterpart’s structure (PDB entry 5mzj, gold). The inset right panel shows details of the ligand-binding pocket for both structures. (*d*) Superposition of the SSX hA_2A_R–LUAA47070 complex structure (PDB entry 8a2p, dark blue) and its synchrotron cryo counterpart’s structure (PDB entry 5olv, light blue). The inset right panel shows details of the ligand-binding pocket for both structures. Ligands, selected lipids and amino acid side chains are in stick representation. Water molecules are shown as spheres and hydrogen bonds are represented by dashes. Pictures were prepared using *PyMOL*. AR3 and hA_2A_R structural alignment RMSD values can be found in Tables S1 and S2, respectively.

The AR3 microcrystals of sizes 5–10 µm taken to DLS were loaded into a high-viscosity injector and passed across the X-ray beam (see Section 2.3) at an average flow rate of ∼5–10 µl min^−1^ at room temperature. From a total sample volume of ∼200 µl of crystal-laden LCP thread [Figs. S1(*a*) and S1(*b*)], 1438 frames were successfully indexed in the space group *P*2_1_2_1_2_1_. The SSX AR3 final structure was solved by molecular replacement and refined to 2.2 Å with an *R*_work_ and *R*_free_ of 22.3 and 25.2%, respectively (Table 1 ▸). The good-quality data and refinement statistics have yielded high-quality electron-density maps [Figs. 2 ▸(*a*) and 2(*e*)] that allowed us to model 43 water molecules and ten lipid fragments. Light-driven proton transporters, such as AR3, critically depend on a coordinated network of internal water molecules to mediate proton translocation across membranes. This hydrogen-bond network in the Schiff base region was clearly resolved in our structure [Fig. 2 ▸(*a*)]. In addition, the retinal chromophore covalently bound to the Lys226 in our SSX AR3 structure (AR3_dark-adapted_) [Fig. 2 ▸(*e*)] was modelled with an occupancy ratio of 70% 13-*cis* and 30% all-*trans* isomers, consistent with previously solved AR3 dark-adapted structures [PDB entry 6gux (Bada Juarez *et al.*, 2021 ▸) and PDB entry 6s63 (Axford *et al.*, 2022 ▸)]. The post-translational modification of Gln7 at the N-terminus (conversion to a pyroglutamate residue) was well resolved (Bada Juarez *et al.*, 2021 ▸). To confirm the validity of the VIALS method, our SSX AR3_dark-adapted_ structure was compared and found to be in good agreement with the dark-adapted structures previously determined at cryo and room temperatures (PDB entries 6gux and 6s63), with Cα RMSD values of 0.25 and 0.24 Å, respectively [Fig. 3 ▸(*a*)]. The minor differences observed were only related to the orientation of a small number of surface side chains. The mean *B* factor of the obtained structure is consistent with the resolution, temperature and data processing (see Table S1).

### 3.2. Room-temperature SFX structure of AR3 obtained 110 ns after photoexcitation

AR3 microcrystals in LCP (5–10 µm) were taken to SACLA and time-resolved SFX data were collected as described in Section 2.4. The LCP thread with a high density of microcrystals was continuously ejected from the high-viscosity sample injector and a femtosecond pump–probe experiment was performed with a time delay of 110 ns. With a crystal hit rate of up to 31% (determined by the SACLA real-time data-processing pipeline; Nakane *et al.*, 2016 ▸), a full data set was obtained within 2.5 h using only ∼60 µl of crystal-laden LCP. A total of 11 912 frames were successfully indexed in the space group *P*2_1_2_1_2_1_ and the high quality of the diffraction data was supported by the figures of merit *R*_split_ and CC_1/2_ of 16.1 and 99.2%, respectively (Table 1 ▸). The AR3_110 ns_ final structure was refined to 1.7 Å resolution with an *R*_work_ and *R*_free_ of 18.12 and 19.64%, respectively. The resulting electron-density maps revealed the presence of 61 water molecules and lipid fragments. Similar to its SSX counterpart structure AR3_dark-adapted_ (PDB entry 6guy), the pentagonal hydrogen-bonding network, formed by the side chains of Asp95 and Asp222 and W402, W401 and W406, was exceptionally well resolved [Fig. 2 ▸(*b*)]. The retinal chromophore was modelled in the all-*trans* state [Fig. 2 ▸(*f*)]. The final SFX AR3_110 ns_ high-quality refined model superimposed very well with both AR3 cryo-cooled (PDB entry 6s6c; Bada Juarez *et al.*, 2021 ▸) and room-temperature SSX (PDB entry 6guz; J. F. Bada Juarez, P. J. Judge, J. Vinals, D. Axford, J. Birch, P. Aller, A. Butryn, R. L. Owen, D. A. Sherrell, J. H. Beale, A. M. Orville, A. Watt & I. Moraes, to be published) light-adapted structures, with Cα RMSD values of 0.18 and 0.09 Å, respectively [Fig. 3 ▸(*b*)]. The mean *B*-factor value of 36.0 Å^2^ is also within the values reported for a room-temperature structure of 1.7 Å (Table S1).

The crystals taken to SACLA were first tested for isomorphism and diffraction quality at the I24 beamline (DLS). On the basis of previous observations and discussions by the SX community (Andersson *et al.*, 2019 ▸; Mehrabi *et al.*, 2021 ▸; Nango *et al.*, 2019 ▸), we postulate that the difference in resolution observed between the SSX AR3_dark-adapted_ and the SFX AR3_110 ns_ structures might be due to the different parameters of the synchrotron beamlines compared with the XFELs. In addition, during our experiments, the extruder nozzle used at SACLA was 75 µm ID whereas that at DLS was 100 µm ID (the only size available at the time). Larger LCP extruder nozzle sizes usually result in data with higher scattering background from the LCP (Andersson *et al.*, 2019 ▸; Kovácsová *et al.*, 2017 ▸; Kubo *et al.*, 2017 ▸). Finally, the SACLA X-ray beam spot dimensions were 1.3 × 1.5 µm (FWHM) while the beam at DLS was focused at 9 × 6 µm (FWHM) for an average crystal size of 5 to 15 µm.

### 3.3. Room-temperature structures of hA_2A_R in complex with theophylline and LUAA47070 by SSX

**Figure 4:**
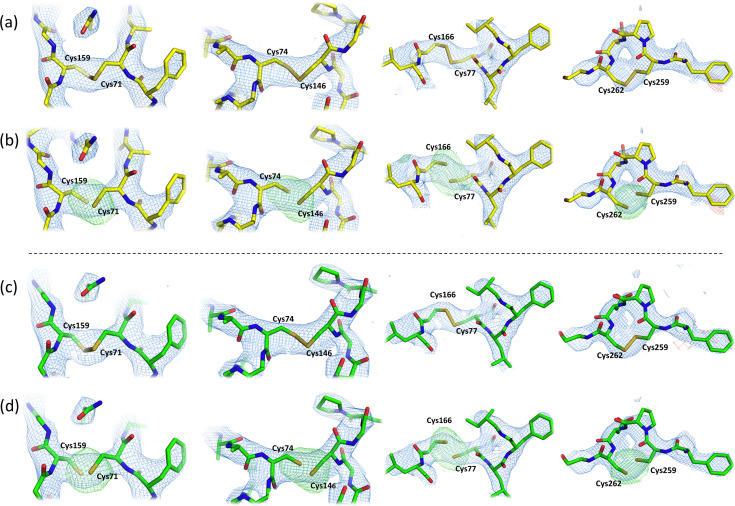
Electron-density maps of hA_2A_R’s four di­sulfide bridges (Cys71–Cys159, Cys74–Cys146, Cys77–Cys166 and Cys259–Cys262). (*a*) and (*c*) show the 2*mF*_o_ − *DF*_c_ (blue mesh, contoured at 1.5σ) and *mF*_o_ − *F*_c_ (green and red meshes, contoured at ±3σ) electron-density maps around the hA_2A_R four di­sulfide bonds in complex with theophylline and LUAA47070, respectively. (*b*) and (*d*) show the omit 2*mF*_o_ − *DF*_c_ (blue mesh, contoured at 1.5σ) and *mF*_o_ − *DF*_c_ (green and red meshes, contoured at ±3σ) electron-density maps when the di­sulfide bridges are omitted/broken. Here, strong positive electron density is seen where original di­sulfide bridges were formed. The hA_2A_R residues in complex with theophylline and LUAA47070 are represented by yellow and green sticks, respectively. Pictures were prepared in *PyMOL*.

To demonstrate the applicability of our VIALS method to structure-based drug design, we performed ligand-soaking experiments. Glass vials containing the hA_2A_R microcrystals were divided into two groups (the same number as the number of ligands) and the crystallization buffer was exchanged (using a gas-tight Hamilton syringe with a long needle) [Fig. 1 ▸(*b*)] with new mother liquor, supplemented either with 0.5 m*M* theophylline, a weak non-selective hA_2A_R antagonist (Segala *et al.*, 2015 ▸), or 1 m*M* LUAA47070, a known hA_2A_R antagonist (Sams *et al.*, 2011 ▸). The crystals within the glass vials were left to incubate at 20°C for 3 to 6 h, to allow ligand exchange and binding, prior to SSX data collection at DLS on the I24 beamline. Using the high-viscosity LCP extruder (Weierstall *et al.*, 2014 ▸) diffraction data were collected at an average flow rate of ∼3–8 µl min^−1^. A total of 10 457 and 3618 frames were successfully indexed in the space group *C*222_1_ for the hA_2A_R in complex with theophylline and LUAA47070, respectively. The final refined structures of hA_2A_R in complex with theophylline and LUAA47070 were refined to 3.45 and 3.50 Å resolution with an *R*_work_/*R*_free_ of 21.85/24.12 and 21.20/25.40, respectively. Despite the resolution, our hA_2A_R SSX structures in complex with theophylline and LUAA47070 were well resolved with good-quality electron-density maps. The presence of the ligands was initially observed by strong 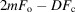 electron densities around the binding pocket and confirmed by difference electron-density omit maps, generated from the crystallographic data [Figs. 2 ▸(*c*), 2(*g*), 2(*d*) and 2(*h*)]. The binding modes of theophylline and LUAA47070 are the same as those observed in the cryo structures (PDB entries 5mzj and 5olv, respectively). In the hA_2A_R–theophylline complex, the ligand forms hydrogen bonds to Asn253 and interacts with Val84, Phe168, Met177, Leu249, Met270 and Ile274 through hydro­phobic interactions [Fig. 3 ▸(*c*)], while in the hA_2A_R–LUAA47070 complex, the antagonist interacts with Tyr9, Glu169, Asn253 and His278 through water-mediated contacts and with Trp246 via van der Waals contacts [Fig. 3 ▸(*d*)]. None of hA_2A_R’s four di­sulfide bonds (Cys71–Cys159, Cys74–Cys146, Cys77–Cys166 and Cys259–Cys262) were broken or showed radiation damage in our room-temperature hA_2A_R complex structures, as suggested by the absence of negative electron-density peaks around the bonds and by omit *mF*_o_ − *DF*_c_ maps (Fig. 4 ▸).

Finally, our structures superimposed very well with those acquired under cryo conditions [PDB entry 5mzj (Cheng *et al.*, 2017 ▸) and PDB entry 5olv (Rucktooa *et al.*, 2018 ▸)], with RMSD values of 0.44 and 0.33 Å for all Cα atoms, respectively. The observed higher average *B* factors were expected for resolutions of 3.5 Å and room-temperature SSX data collection (Table S2).

## 4. Summary

VIALS (versatile approach to high-density microcrystals in lipidic cubic phase for serial crystallography) is a semi-automated high-throughput method for the large-scale production of microcrystals of membrane proteins in LCP. The method allows a more efficient screening of crystallization conditions than is possible with the routinely used glass syringe method, and is suitable for ligand soaking for drug-design studies. Finally, it has the advantage of easy sample transportation compared with syringes. We have demonstrated the compatibility of the microcrystals generated with LCP injectors at both synchrotron and XFEL sources. Room-temperature structures of the AR3 photoreceptor protein in its dark-adapted state and of the 110 ns photocycle intermediate were obtained, and the first room-temperature structures of hA_2A_R in complex with theophylline and LUAA47070 were solved. We also anticipate that the microcrystals generated will be suitable for heavy-atom soaking *in meso.*

## Supplementary Material

Supporting figures and tables. DOI: 10.1107/S1600576723006428/jt5069sup1.pdf

PDB reference: room-temperature structure of the stabilized A2A–LUAA47070 complex determined by synchrotron serial crystallography, 8a2p

PDB reference: room-temperature structure of the stabilized A2A–theophylline complex determined by synchrotron serial crystallography, 8a2o

PDB reference: room-temperature structure of archaerhodopsin-3 dark-adapted, 6guy

PDB reference: room-temperature structure of archaerhodopsin-3 obtained 110 ns after photoexcitation, 7zy3

## Figures and Tables

**Table 1:** Crystallographic statistics for data collection Numbers in parentheses refer to the highest-resolution shell.

	SSX_AR3_dark-adapted_	TR-SFX_AR3_110 ns_	hA_2A_R–theophylline	hA_2A_R–LUAA47070	
Data collection					
X-ray source	I24/DLS	BL2 EH3, 4b/SACLA	I24/DLS	I24/DLS	
Detector	Dectris PILATUS3 6M	4MPCCD	Dectris PILATUS3 6M	Dectris PILATUS3 6M	
Temperature (K)	293	293	293	293	
Extruder nozzle (µm)	100	75	50	50	
Wavelength (Å)	0.96862	1.24 (10.0 keV)	0.96862	0.96862	
Beam size (µm)	9 × 6	1.3 × 1.5	7 × 7	7 × 7	
No. of frames	1438	11912	10457	3618	
Space group	*P*2_1_2_1_2_1_	*P*2_1_2_1_2_1_	*C*222_1_	*C*222_1_	
Cell dimensions					
*a*, *b*, *c* (Å)	46.29, 48.27, 104.90	46.20, 48.30, 104.70	40.53, 182.31, 144.27	40.44, 181.84, 144.64	
α, β, γ (°)	90, 90, 90	90, 90, 90	90, 90, 90	90, 90, 90	
Resolution range (Å)	43.9–2.2 (2.24–2.20)	28.29–1.70 (1.72–1.70)	91.16–3.45 (3.52–3.45)	90.93–3.50 (3.56–3.50)	
No. of unique observations	12478 (600)	26515 (1299)	7416 (346)	7126 (344)	
Completeness (%)	100 (100)	100 (100)	99.4 (96.90)	99.6 (100)	
Multiplicity	31.3 (14.20)	101.5 (65.2)	233.6 (17.30)	88.9 (36.00)	
*R*_split_	0.200 (0.762)	0.161 (1.820)	0.168 (0.955)	0.220 (0.641)	
CC_1/2_	0.9372 (0.535)	0.992 (0.636)	0.985 (0.236)	0.973 (0.595)	
Mean *I*/σ(*I*)	2.16 (0.46)	3.79 (0.59)	20.64 (1.48)	13.68 (1.98)	
Wilson *B* factor (Å^2^)	29.40	32.3	88.0	84.1	

Refinement					
Resolution range (Å)	28.3–2.20 (2.24–2.20)	28.15–1.70 (1.77–1.70)	23.63–3.45 (3.54–3.45)	23.58–3.50 (3.59–3.50)	
No. observations (total/test set)	12469/1247	26493/1311	7034/370	6398/696	
Completeness (%)	99.90 (99.90)	99.95 (99.80)	99.45 (98.31)	100 (100)	
*R*_work_/*R*_free_ (%)	22.30/25.20	18.12/19.64	21.85/24.12	21.20/25.40	
No. of atoms					
Protein	1894	1980	2992	3008	
Ligand/ion	162	103	176	164	
Waters	43	61	20	12	
Average *B* all atoms (Å^2^)	39	36	129	118	
R.m.s. deviations					
Bond lengths (Å)	0.003	0.006	0.002	0.008	
Bond angles (°)	0.585	0.809	0.638	1.466	

Ramachandran plot					
Outliers (%)	0.0	0.0	0.0	0.0	
Allowed (%)	2.52	0.84	2.40	1.63	
Favoured (%)	97.48	99.16	97.60	98.37	
					
PDB code	6guy	7zy3	8a2o	8a2p	
