# Supplementary material for: A versatile approach to high-density microcrystals in lipidic cubic phase for room-temperature serial crystallography
Source: J Appl Crystallogr. 2023 Aug 18;56(Pt 5):1361–70. doi: 10.1107/S1600576723006428 (PMC10543674; doi:10.1107/S1600576723006428)
Supplement: Supplementary file 1 [file j-56-01361-sup1.pdf]

JOURNAL OF APPLIED CRYSTALLOGRAPHY

Volume 56 (2023)

Supporting information for article:

A versatile approach to high-density microcrystals in lipidic cubic phase for room-temperature serial crystallography

James Birch, Tristan O. C. Kwan, Peter J. Judge, Danny Axford, Pierre Aller, Agata Butryn, Rosana I. Reis, Juan F. Bada Juarez, Javier Vinals, Robin L. Owen, Eriko Nango, Rie Tanaka, Kensuke Tono, Yasumasa Joti, Tomoyuki Tanaka, Shigeki Owada, Michihiro Sugahara, So Iwata, Allen M Orvile, Anthony Watts and Isabel Moraes

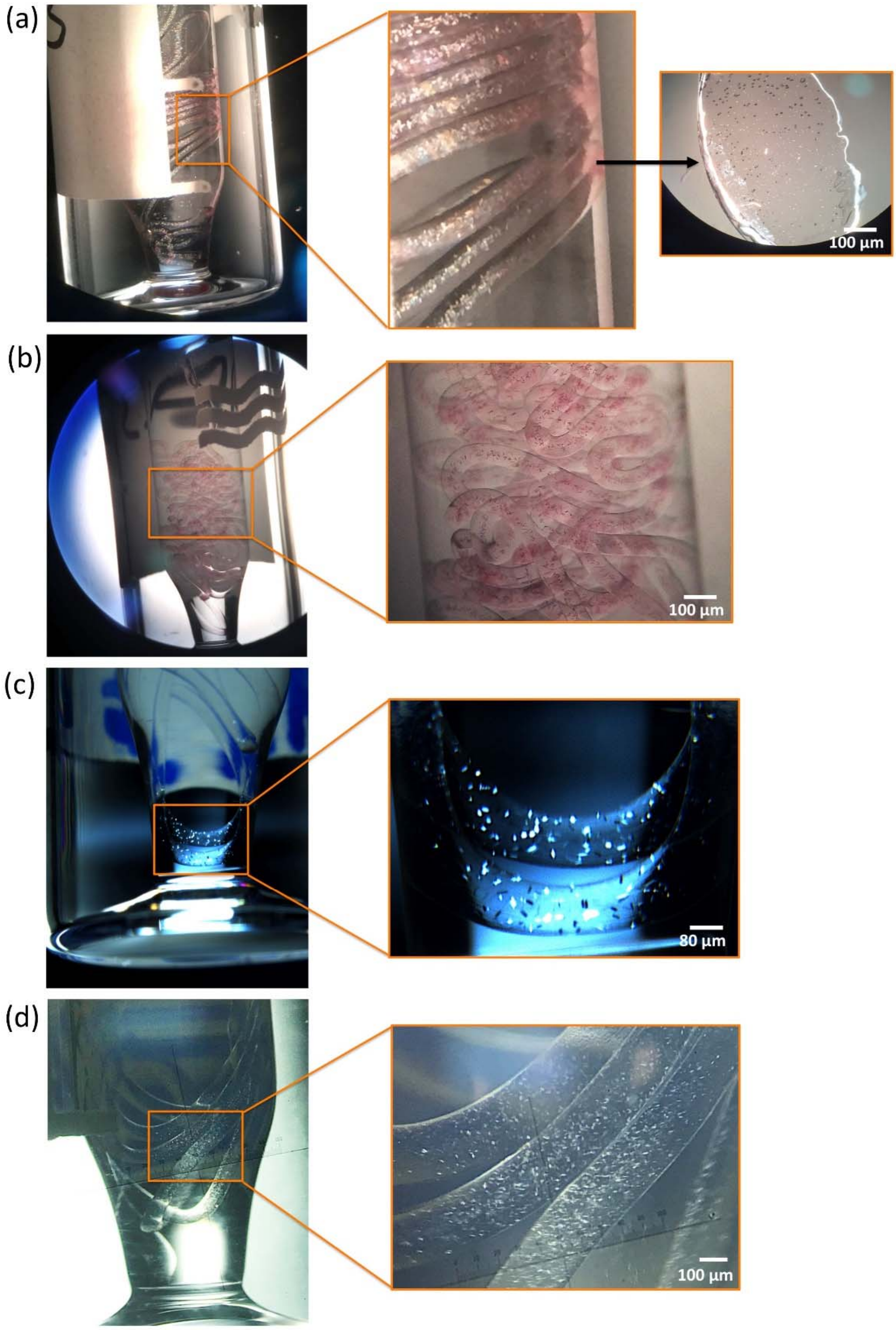

Figure S1 Microscope images of membrane protein microcrystals grown in lipidic cubic phase for serial crystallography measurements using the VIALS method. (a) Bright field of Archaerhodopsin-3 (AR3) microcrystals inside the glass vials (left) and sandwiched between two glass slides (b) Bright

field images of AR3 microcrystals. (c) Cross-polarized images of $hA_{2A}R$ microcrystals soaked in theophylline (d) Bright field images of $hA_{2A}R$ microcrystals soaked in LUAA47070.

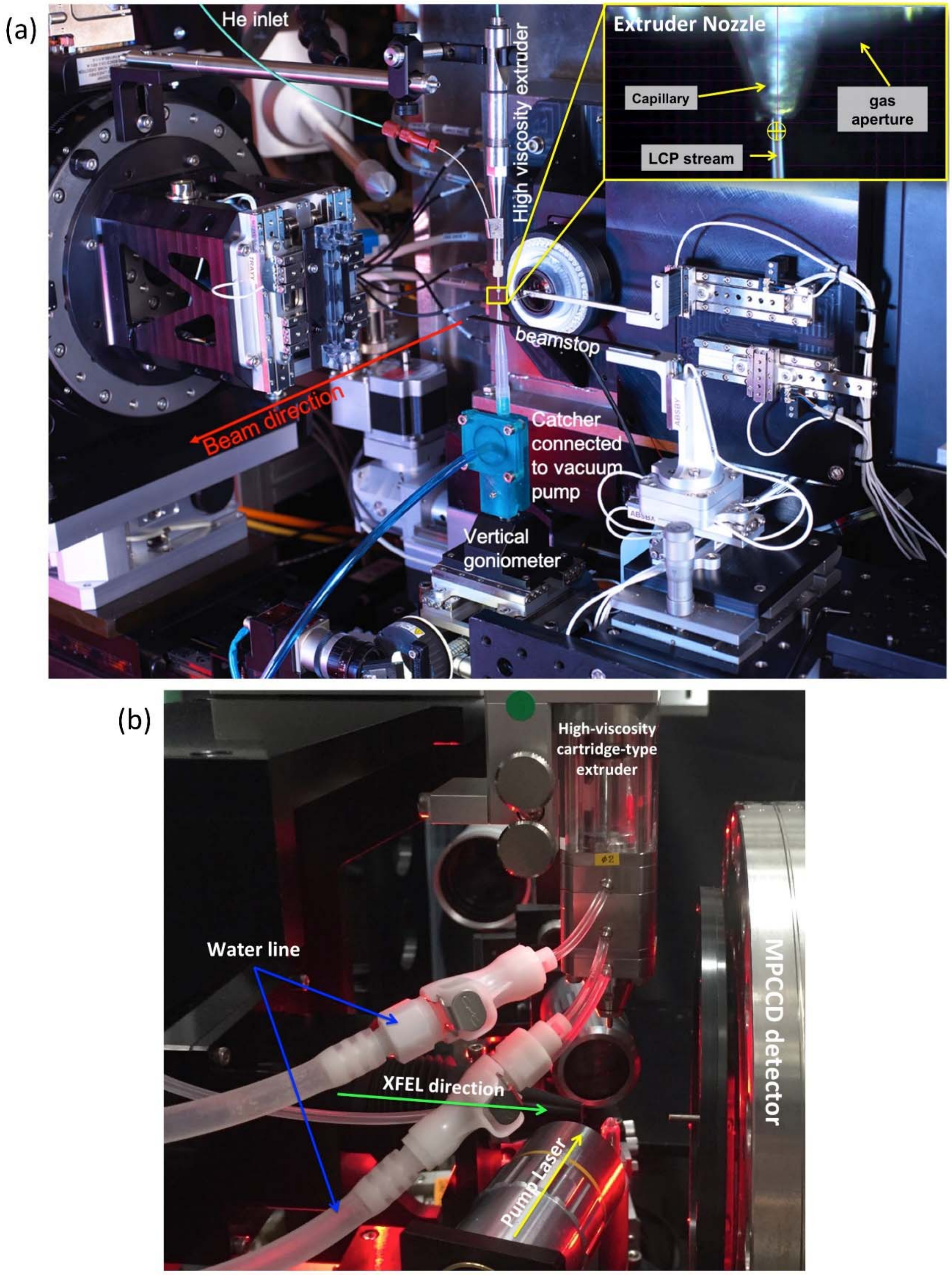

Figure S2 End-station setup for serial crystallography data collection from microcrystals in LCP. (a) End-station of I24 beamline at Diamond Light Source (UK) in serial synchrotron crystallography

mode using a high-viscosity LCP extruder mounted vertically (top centre) and aligned with a catcher connected to a vacuum pump for collection of the exposed LCP sample. Co-flowing He gas is used for reliable extrusion and sample coaxial flow (inset) (Weierstall *et al.*, 2014). (b) Endstation set up at BL2 EH3 4b beamline at SACLA during pump probe serial femtosecond crystallography data collection. Microcrystals in LCP are loaded and delivered at room temperature using a HVC extruder. A coaxial gas flows along the inner capillary in the nozzle keeps the LCP stream on-axis. The pump ns-laser, to photoactivate the protein at set delayed times, operates at half repetition rate of that of XFEL and illuminates both sides of the LCP extruder to maximize excitation efficiency. A vacuum pump to collect the residual LCP sample is placed directly below the HVC nozzle (Shimazu *et al.*, 2019; Tono *et al.*, 2015).

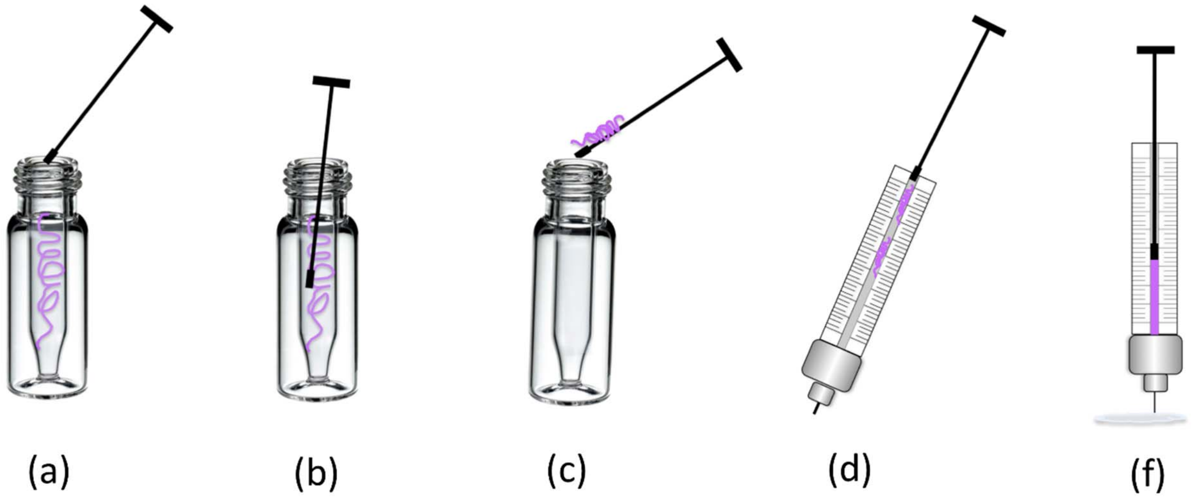

Figure S3 Diagram depicting the procedure used to retrieve the microcrystals in the LCP threat from the glass vials. (a), (b) and (c) With the help of a syringe plunger (a thin metal rod can also be used) the microcrystals in the LCP threat were easily and fast (to avoid much dehydration) removed from the glass vials and transferred to (d) a 250 µl gas-tight Hamilton syringe (No. 81120 Hamilton). The procedure was repeated until enough LCP to fill the extruder reservoir for the experiment. (f) Once the required LCP quantity was transferred, slowly with the help of the syringe plunger, the LCP thread was pushed forward to release any excess of precipitant. The LCP extruders were loaded as recommended by their developers (Weierstall *et al.*, 2014; Shimazu *et al.*, 2019).

Supplementary Table 1 – C_α r.m.s.d. values for comparison between the cryo, SSX and SFX AR3 structures.

AR3 Dark Adapted		
**Cryo-cooled**<br>(Single crystals)<br><br>PDB: 6GUX<br>Resolution: 1.30 Å<br>Mean *B* factor: 17.64 Å^2	**RT-SSX**<br>(LCP-extruder)<br><br>PDB: 6GUY<br>Resolution: 2.20 Å<br>Mean *B* factor: 39.6 Å^2	**RT-SSX**<br>(Thin-film)<br><br>PDB: 6S63<br>Resolution: 1.85 Å<br>Mean *B* factor: 26.54 Å^2
**r.m.s.d**<br>0.25 Å (C_α atoms)		**r.m.s.d**<br>0.24 Å (C_α atoms)
**AR3 Light Adapted & photocycle intermediate**		
**Cryo-cooled**<br>(Single crystals)<br><br>PDB: 6S6C<br>Resolution: 1.07 Å<br>Mean *B* factor: 17.97 Å^2	**TR-SFX**<br>(SACLA LCP-extruder)<br><br>PDB: 7ZY3<br>Resolution: 1.70 Å<br>Mean *B* factor: 36.0 Å^2	**RT-SSX**<br>(Thin-film)<br><br>PDB: 6GUZ<br>Resolution: 1.90 Å<br>Mean *B* factor: 21.78 Å^2
**r.m.s.d.**<br>0.18 Å (C_α atoms)		**r.m.s.d.**<br>0.09 Å (C_α atoms)

Supplementary Table 2 – C_α r.m.s.d. values for comparison between the Cryo, SSX and SFX $hA_{2A}R$ complex (Theophylline and LUAA47070) structures

**$hA_{2A}R$ in complex with Theophylline**		**$hA_{2A}R$ in complex with LUAA47070**	
**Cryo-cooled**<br>(Single crystals)<br><br>PDB: 5MZJ<br>Resolution: 2.0 Å<br>Mean *B* factor: 42.0 Å^2	**RT-SSX**<br>(LCP-extruder)<br><br>PDB: 8A2O<br>Resolution: 3.5 Å<br>Mean *B* factor: 101 Å^2	**Cryo-cooled**<br>(Single crystals)<br><br>PDB: 5OLV<br>Resolution: 2.0 Å<br>Mean *B* factor: 41 Å^2	**RT-SSX**<br>(LCP-extruder)<br><br>PDB: 8A2P<br>Resolution: 3.5 Å<br>Mean *B* factor: 81.0 Å^2
**r.m.s.d.**<br>0.44 Å (C_α atoms)		**r.m.s.d.**<br>0.33 Å (C_α atoms)	
